# Association of *Neospora caninum* with reproductive performance in dairy cows: A prospective study from Iran

**Published:** 2017-06-15

**Authors:** Maryam Ansari-Lari, Abbas Rowshan-Ghasrodashti, Hadi Jesmani, Maliheh Masoudian, Mehrdad Badkoobeh

**Affiliations:** 1 *Department of Food Hygiene and Public Health, School of Veterinary Medicine, Shiraz University, Shiraz, Iran;*; 2 *Department of Internal Medicine, School of Veterinary Medicine, Islamic Azad University, Kazeroon Branch, Kazeroon, Iran; *; 3 *Department of Clinical Studies, School of Veterinary Medicine, Shiraz University, Shiraz, Iran; *; 4 *Department of Pathobiology, School of Veterinary Medicine, Shiraz University, Shiraz, Iran.*

**Keywords:** Abortion, Dairy cow, Iran, *Neospora caninum*, Reproduction

## Abstract

There is no prospective study from Iran to estimate the direct risk of *Neospora caninum* for pregnancy loss or reproductive factors. In addition, there is no report in the literature concerning the association of *N. caninum* with dystocia and sex of calves. Therefore, this study was conducted on a group of dairy cows in a large intensive production system during 2011 to 2013 in southern Iran to evaluate the impact of neosporosis on reproductive performance. A total of 253 cows which were diagnosed as pregnant during the first six months of the study were followed until calving or abortion. Reproductive data were collected and *N. caninum* serostatus was determined using ELISA. To investigate the association of abortion with* N. caninum*, survival analysis was performed using Cox proportional hazard model. The *N. caninum* seroprevalence in the study group was 30.40% (95% CI: 27.40, 36.10). The overall abortion rate was 12.25%, significantly higher in seropositive animals (20.80%) than seronegative ones (8.50%). Results of Cox model showed that serostatus of animal for *N. caninum *and season had significant associations with abortion (*p *< 0.01). *Neospora caninum* did not show significant association with other factors such as dystocia and sex of calves. In conclusion, neosporosis is responsible for 12.00% excess abortion risk in infected group and more than 30.00% of abortions could be preventable by control of *Neospora* in study population. Therefore, control of *N. caninum *would reduce the economic losses caused by parasite mainly due to pregnancy loss.

## Introduction

*Neospora caninum*, an apicomplexan parasite, is an obligate intracellular parasite of animals with a wide host range. It is now regarded as a major cause of bovine abortion with negative economic and reproductive impacts in the cattle industry.^1^ The economic impacts of infection with *N.* *caninum *in cattle herds include costs associated with abortion,^2^ increased number of culled cows^3^ and decreased milk production.^4^ Although, there are some reports that milk production may increase in seropositive cows.^5,6^

In Iran, there are several reports from various parts of the country about the seroprevalence of *N. caninum *antibodies in dairy cattle farms.^7-13^ A few studies showed the association between *N. caninum *and abortion in dairy cows through evaluation of aborted fetuses for diagnosis of infection.^14-17^ However, no prospective epidemiological study has been performed in the country to estimate the direct risk of abortion and evaluate the reproductive indices in infected cows. In addition, there is no report in the literature concerning the association of *N. caninum* with dystocia and sex of calves.

 Knowledge of reproductive performance among infected and non-infected cows in each region would increase our understanding of the economic impacts of the infection and probable benefits anticipated if *N. caninum *infection was eradicated from a herd. Therefore, the present study was conducted in Shiraz, the capital of Fars province, southern Iran to evaluate the impact of neosporosis on abortion as well as reproductive indices including calving to conception interval, calving interval and number of services per conception. Furthermore, sex of calves, occurrence of retained placenta and dystocia in dairy cattle were investigated in this study.

## Materials and Methods

**Study herd. **This prospective study was conducted on a group of dairy cows in a large intensive production system in Shiraz during late 2011 to early 2013. The study farm was a Holstein dairy herd milking 1,200 cows; tuberculosis and brucellosis free, with non-seasonal (year-round) calving program and the cows were artificially inseminated as a routine. However, no vaccinations for infectious bovine rhinotracheitis or bovine viral diarrhea were performed in this herd. Cows were housed in open-shed barns, milked three times a day and the milk was used for manufacturing. Their rations were based primarily on corn silage, alfalfa hay and some concentrates with ground barley and corn as the main energy sources. Artificial insemination was done by trained persons and diagnosis of pregnancy was performed by ultrasound devices between 32 and 38 days post-insemination with follow-up examinations on day 60 and day 210. The farm had reliable and regular record keeping and veterinary consultant for disease and nutrition management. 

**Study animals and data collection. **A total of 253 cows which were diagnosed as pregnant during the first six months of the study were followed until calving or abortion. Blood samples were collected from the study group at early gestation months and sera were stored at –20 ˚C until use. Data about date of birth (for calculating the age of animal), parity, previous abortion, number of services per conception, calving to conception interval, calving interval, calving status (dystocia and retained placenta) and sex and status of calf (stillbirth or not) were collected for the study group. Date of last service was considered as the date of conception. Calving to conception interval was calculated as the interval between date of last service and date of previous calving. For calculation of calving interval and calving to conception interval, aborted animals were excluded from the analysis. Abortion was defined as fetal loss after confirmation of pregnancy. Age of abortion was estimated based on the date of conception and date of observed obvious abortion or the date of first negative pregnancy test in the follow up examination. 

**Serological examination. **For serological examination and detection of *N. caninum* antibodies, a commercial ELISA kit (IDEXX Laboratories Inc., Westbrook, USA) was used. All sera were diluted 1:100 in phosphate buffered saline solution and according to the instruction of the manufacturer; all samples with an absorbance value above the cut-off level of 0.50 were considered as positive.

**Statistical analysis. **For statistical examination, SPSS statistical software (version 16; SPSS Inc., Chicago, USA) was used. Continuous data were presented as mean, standard deviation (SD) and median and categorical variables were displayed as number and percent. For comparison of continuous variables between* N. caninum* positive and negative groups, two independent samples t-test was used. Association of *Neospora* status with abortion and other pregnancy outcomes was investigated using Chi-square analysis or Fisher’s exact test. In addition, to investigate the association of abortion with* N. caninum *with respect to time of abortion, survival analysis was performed using Cox proportional hazard model. Abortion was considered as failure event and age of abortion or calving (days gestation) was time of event. Six animals were sold before calving due to financial needs. They were considered as censored cases in the Cox model. Serological status of animal for *N. caninum*, season of abortion (summer versus other seasons), parity (primiparous versus multi-parous) and history of abortion in previous pregnancy were included as covariates in the model. To investigate the possible interaction between *N. caninum* serostatus and season, the interaction term also introduced to model. Using backward elimination procedure for non-significant factors, the final Cox model was constructed. To check the proportional-hazards assumption, graphical methods were

 used: the log of the negative log of survival was plotted against days gestation with the significant independent variables as strata. No violation of the proportionality assumption was evident. In all analyses, a *p*-value less than 0.05 was considered as statistical significance. 

## Results

Results showed that 77 out of total 253 cows were positive for *N. caninum *based on serological examination. The overall seroprevalence in the study group was 30.40% (95% CI: 27.40, 36.10). The mean age of all animals was 4.70 and SD was 2 years. Age, parity, calving to conception interval and calving interval of animals based on their serological status for *N. caninum *are shown in Table 1. No significant differences were observed between groups (*p *> 0.05). 

The outcomes of pregnancy as well as number of services per conception in two categories (1 and >1) for the study group are presented in Table 2. The overall abortion rate was 12.25% (95% CI: 8.20-16.30). The incidence of abortion in the seropositive group was 20.80% (16 out of total 77), significantly higher than 8.50%, (15 out of total 176) in the sero-negative group (*p *= 0.006). Attributable risks in the exposed group and in the population were 12.00% and 3.70%, respectively. Attributable fraction in the population was 30.00%. Number of services per conception and occurrence of dystocia, retained placenta, stillbirth and sex of calves were not different between *N. caninum *positive and negative groups (*p *> 0.05). 

Mean age of abortion and SD in seropositive cows were 4.30 and 1.50 months (129 ± 46 days), respectively. The corresponding measures for seronegative cows were six and two months (183 ± 76 days), respectively. There was significant difference between two groups (*p *= 0.02). Seven cases of aborted cows had history of abortion in their previous pregnancies. 

A total of 3.20% of abortions (1 out of 31 cases) and 14.30% of *Neospora* positive cows (11 out of 77 cases) were heifers (first parity). No association was observed between parity of animal and its *Neospora* serostatus (*p *= 0.35) or abortion (*p *= 0.12). Statistically significant more abortions (18 out of 31, 58%) occurred during the summer months compared with other seasons (*p *< 0.001).

**Fig. 1:**
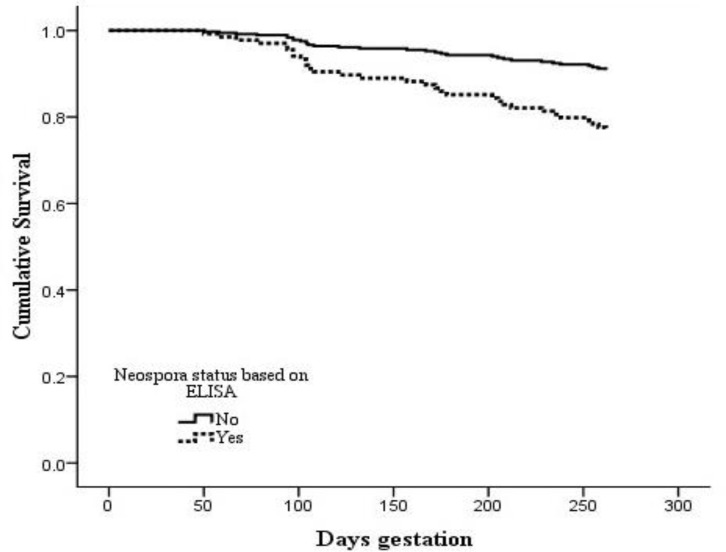
Survival plot for age of abortion comparing *N. caninum *positive and negative cows

Figure 1 shows the survival curve for age of abortion or calving according to *Neospora* status of cows. Sero positive animals had more than two fold greater hazard (HR = 2.50, 95% CI: 1.22, 5.04, *p *= 0.012) of abortion than seronegative cows. Season was significant in the model and risk of abortion was 6.90 (95% CI for HR: 3.30, 14.31) fold higher in summer months compared with other seasons (*p *< 0.001). Also, cows with history of previous abortion had two fold (HR=2.30, 95% CI: 0.98, 5.56, *p *= 0.055) more chance of abortion than other cows. Parity (*p *= 0.26) and interaction term for season × *Neospora* serostatus (*p *= 0.73) were not significant and excluded from the model. 

**Table 1:** Summary measures for cows based on their serological status for *N. caninum. *Data are presented as mean ± SD

**Parameters**	**Seropositive**	**Seronegative**	***p*** **-value**
**Age (Years)**	4.70 ± 2.10	4.70 ±1.90	0.87
**Parity**	3.00 ± 1.70	3.10 ±1.50	0.82
**Calving to conception i** **nterval (Days)** *	147.00 ± 94.00	134.00 ±84.00	0.31
**Calving interval (Days)** *	434.00 ± 101.00	412.00 ±84.00	0.13

* Aborted animals were excluded from the analysis.

**Table 2:** Outcome of pregnancy in cows based on their serological status for *N. caninum*

**Reproductive parameter**		**Number of** **seropositive (%)**	**Number of** **seronegative (%)**	**Total**	***p*** **-value**
**Abortion**	Yes	16 (20.80)	15 (8.50)	31 (12.30)	0.006
	No	61 (79.20)	161 (91.50)	222 (87.70)	
**No. of services per conception**	1	26 (33.80)	69 (39.20)	95 (37.50)	0.41
	>1	51 (66.20)	107 (60.80)	158 (62.50)	
**Dystocia** a	Yes	14 (24.00)	34 (22.70)	48 (23.10)	0.82
	No	44 (76.00)	116 (74.30)	160 (76.90)	
**Retained placenta** a	Yes	4 (7.10)	13 (8.70)	17 (8.30)	0.20
	No	52 (92.90)	136 (91.30)	188 (91.70)	
**Sex of calves ** a b	Male	33 (56.90)	72 (46.50)	105 (49.30)	0.17
	Female	25 (43.10)	83 (53.50)	108 (50.70)	
**Stillbirth** ^a^	Yes	3 (5.00)	5 (3.00)	8 (4.00)	0.62
	No	57 (95.00)	154 (97.00)	211(96.00)	

a There is a few missing data for some of these variables due to non-recording or inaccurate recording.

b A few twin pregnancies were included in analysis.

## Discussion

The present work is the first prospective study in the country to determine the reproductive performance of a group of naturally infected cattle with *N. caninum* in a large intensive production system in the region. The result of our study showed that incidence of abortion is significantly higher in *N. caninum *positive cows than their negative herd mates. However, the majority of sero-positive cows (79.00%) had a normal pregnancy which is in agreement with previous reports.^18^ In our study, hazard of abortion was 2.50 fold higher in seropositive cows than seronegative ones. This is in the range of previously reports which indicated that* N. caninum *could increase the odds of abortion 1.70 to 7.20 fold in dairy cows.^2^ Considering the results, neosporosis is responsible for 12.00% excess abortion risk in infected group in the present study and more than 30.00% of abortions could be preventable by control of *Neospora* in the study population. In Iran, no inclusive efforts in management practices are performed regarding control and prevention of neosporosis in dairy cattle. One reason may be the fact that so far, no direct estimate of the risk of abortion in seropositive cows in the country was available. In each region, there are some differences in herd structure and management practices. The results of the present study are confined to one industrial dairy herd and generalization of the results might be limited. However, as noted above, this herd is a large dairy herd with regular record keeping and good management practices. Thus, the impact of neosporosis on reproduction factors, mainly pregnancy loss in the present study could be assumed as minimum or baseline loss. The economic losses would be expected to be equal or greater in other herds in accordance with their level of management practices. Considering the high rate of abortion in infected cows (20.80%) and substantial population attributable fraction (30.00%) of this parasitic infection for abortion in dairy cows, it could be the time to implement vigorous educational followed by prevention and control programs in our region. 

Following recrudescence of infection, a fetus is exceptionally vulnerable to *N. caninum* infection during early gestation (e.g. < 100 days gestation) when the thymus, spleen and peripheral lymph nodes are immature. However, most abortions are reported to occur during mid-gestation e.g. 100 to 150 days.^18^ This is in agreement with the results of present study which mean ages of abortion in seropositive cows was 129 days; significantly lower than abortion age in seronegative cows (183 days). 

We observed that history of previous abortion is associated with two fold abortion risk. The results were close to significance and in agreement with previous reports indicating that *Neospora* seropositivity can be very stable through time and infected cows can show a variable rate of repeat abortions.^19^ Also, cows with a history of previous abortion were more likely to abort than cows with no prior history of abortion.^20^ Hence, culling of seropositive animals with one abortion could be considered as an option for limiting the economic losses due to this parasitic infection.

It has been reported that abortions due to* N. caninum* occur year-round.^1^ In the present study, most abortions (58.00%) occurred during the summer months compared with other seasons. The stressful conditions due to high temperature may play a role in occurrence of abortion generally and in recrudescence of infection and consequent abortion in a seropositive dam. 

There was no report in the literature concerning the association of *N. caninum* with dystocia and sex of calves. However, the results for stillbirth are inconsistent. In a Canadian study which examined the long-term impact of a *N. caninum*-associated abortion outbreak in a large cow-calf herd in northern Alberta, no association with increased risk of stillbirth was detected.^21^ Meanwhile, a tendency of having more stillbirths was reported in a study from Estonian dairy herds.^22^ Retained placenta was investigated in a recent work which indicated that seropositive cows present greater occurrence of retained placenta.^23^ Based on the present work, no significant difference was observed between seropositive and seronegative groups for dystocia, retained placenta, stillbirth and sex of calves. 

There are few reports investigating the reproductive factors including calving interval, calving to conception interval and number of services per conception in seropositive cows and the existing works show conflicting results. Two recent works observed a negative impact of infection, a study by Pessoa *et al.* reported a longer interval from parturition or abortion to conception in seropositive compared with seronegative cows in grazing lactating dairy cows and another by O’ Doherty *et al.* indicated that exposure to *N. caninum* is associated with compromised reproductive performance.^23^^,^^24^

Some authors through a cross-sectional study in Costa Rica indicated that *Neospora* serostatus does not have a significant effect on the length of calving interval and number of services per conception.^25^ Also, no significant association of *Neospora* with calving interval was observed in a random sample of herds or in herds with history of epidemic-abortion in Dutch dairy herds.^26^ Interestingly, authors in another study reported that herds with at least one seropositive cow have lower odds ratio of higher calving intervals.^22^ In this study, calving to conception interval, calving interval and number of services per conception were not significantly different in seropositive cows compared with seronegative dams. It seems that more researches about the association of reproductive factors (other than abortion) along with a meta-analysis in future could result in a consensus in this context. 

In conclusion, *N. caninum *is an important abortifacient factor in dairy cattle in Shiraz like other parts of the world. However, no effect of neosporosis on other reproductive factors was observed in this study. Nevertheless, it is the time to start implementation of educational, preventive and control programs to reduce the economic impacts of this parasite which are mainly due to pregnancy loss. 
